# A Multilevel Model Approach for Investigating Individual Accident Characteristics and Neighborhood Environment Characteristics Affecting Pedestrian-Vehicle Crashes

**DOI:** 10.3390/ijerph17093107

**Published:** 2020-04-29

**Authors:** Seunghoon Park, Dongwon Ko

**Affiliations:** 1 Department of Urban Planning, Keimyung University, Daegu 42601, Korea; 2 Gyeonggi Research Institute, Suwon 16207, Korea; dw2774@naver.com

**Keywords:** pedestrian-vehicle crashes, built environment, multilevel model, pedestrian injury severity

## Abstract

Walking is the most basic movement of humans and the most fundamental mode of transportation. To promote walking, it is necessary to create a safe environment for pedestrians. However, pedestrian-vehicle crashes still remain relatively high in South Korea. This study employs a multilevel model to examine the differences between the lower-level individual characteristics of pedestrian crashes and the upper-level neighborhood environmental characteristics in Seoul, South Korea. The main results of this study are as follows. The individual characteristics of pedestrian-vehicle crashes are better at explaining pedestrian injury severity than built environment characteristics at the neighborhood level. Older pedestrians and drivers suffer more severe pedestrian injuries. Larger vehicles such as trucks and vans are more likely to result in a high severity of pedestrian injuries. Pedestrian injuries increase during inclement weather and at night. The severity of pedestrian injuries is lower at intersections and crosswalks without traffic signals than at crosswalks and intersections with traffic signals. Finally, school zones and silver zones, which are representative policies for pedestrian safety in South Korea, fail to play a significant role in reducing the severity of pedestrian injuries. The results of this study can guide policymakers and planners when making decisions on how to build neighborhoods that are safer for pedestrians.

## 1. Introduction

From the 1970s, rapid industrialization caused a considerable portion of the South Korean population to migrate to Seoul, its capital city, to seek jobs opportunities, thereby accelerating the process of urbanization of the country [1]; however, a rapid increase in the urban population caused a serious housing crisis [2,3,4]. In response to this crisis, the South Korean government permitted the rapid construction of residential buildings over the recent decades, thereby leading to reckless land development in the suburbs surrounding Seoul; this development resulted in urban sprawl that forced residents to rely on cars. Consequently, pedestrian-vehicle crashes have increased within South Korea’s automobile-oriented society [5].

Hence, to reduce pedestrian-vehicle crashes, the South Korean government has adopted a transportation paradigm that attempts to make its automobile-oriented society safer for pedestrians. It is important for societies to ensure safe walking environments for its citizens, as pedestrians are much more likely to suffer serious injuries from a traffic accident than drivers [6,7,8,9]. In South Korea, various studies have suggested methods for reducing pedestrian-vehicle crashes [10,11,12,13,14,15,16]. Choi et al. [11] insisted on the need to focus on the silver zones for the safety of the elderly. Kwon and Park [14] emphasized the importance of the child pedestrian safety around elementary schools. However, the level of such crashes in South Korea remains high.

In 2016, South Korea had one of the highest rates of fatalities per capita for road traffic accidents among Organization for Economic Co-operation and Development (OECD) countries [17]. In short, a pedestrian’s risk of death or injury is much higher than in other countries. Despite the fact that the number of road fatalities has decreased steadily over the past decade due to improved pedestrian safety policies, the share of pedestrian fatalities among the total number of road fatalities has increased from 35% to 38% [18]; this percentage is much higher than the OECD average of 20% [17].

This study examines individual and neighborhood environmental characteristics that affect the severity of pedestrian-vehicle crashes in Seoul, South Korea. Particularly, this study employs a multilevel model to account for the differences between the lower-level individual characteristics and the upper-level neighborhood environmental characteristics of pedestrian-vehicle crashes. Our findings are significant, as they will allow for more informed discussions on pedestrian safety.

## 2. Literature Review

### 2.1. Individual Characteristics of Pedestrian-Vehicle Crashes

Recent research on pedestrian-vehicle crashes can be largely categorized into studies on the frequency of crashes and studies on the severity of pedestrians’ injuries [19,20,21,22,23,24,25,26,27,28,29]. To create a safe environment for pedestrians, it is important to reduce the frequency of crashes. However, it is equally important to prevent serious pedestrian injuries resulting from pedestrian-vehicle crashes.

The majority of research has focused on particular characteristics of pedestrian crashes that influence the severity of pedestrian injuries. These characteristics have been mainly explored on the basis of three types of characteristics; namely, driver, pedestrian, and crash event characteristics.

Regarding driver characteristics, most studies have revealed that pedestrian injuries are likely to be more severe in pedestrian crashes involving a male driver [15,23,30,31,32]. Moreover, the severity of pedestrians’ injuries has been increased with the increase in the age of the driver [23,31]. However, Pour-Rouholamin et al. [33] analyzed the severity of pedestrian injuries in Illinois, U.S., by comparing drivers under the age of 24 years to those over the age of 65 years and found that these two groups can demonstrate very different results: drivers under the age of 24 years are more likely to cause severe pedestrian injuries compared with those in the age group of 25 to 64 years; however, drivers over the age of 65 years are more likely to cause no/possible pedestrian injuries compared with those in the age group of 25 to 64 years. Choi et al. [34] has also analyzed instances of severe pedestrian injury involving novice drivers and found that less driving experience correlates to more severe pedestrian injuries.

Regarding pedestrian characteristics, some studies have revealed that female pedestrians are more likely to be injured in pedestrian-vehicle crashes than male pedestrians [15,32,35]. However, Ulfarsson et al. [30] stated that male pedestrians are more likely to suffer serious injuries while analyzing the North Carolina regions. Meanwhile, some studies have examined pedestrian accidents on the basis of age [33,36,37]. Most studies have demonstrated that pedestrian injuries are more severe among elderly pedestrians. In particular, Pour-Rouholamin et al.’s study [33] of children-involved crashes shows that pedestrian injury is more serious when children are younger. Chen and Fan [38] also noted that the age of a pedestrian has an effect on the crash fatality rates, but not on other severity levels. In addition, Haleem et al. [39] studied the severity of pedestrian injuries at signalized and non-signalized intersections in Florida, U.S., and stated that very old people involved in crashes at both signalized and non-signalized intersections had similar levels of severe injuries; however, middle-aged individuals were more likely to suffer serious injuries at non-signalized intersections. They argued that it is necessary to not only understand the behavioral characteristics of drivers and pedestrians, but also strengthen traffic regulations to reduce pedestrian fatalities.

Regarding crash event characteristics, the weather has also been found to affect the severity of pedestrian injuries. The severity of pedestrian injuries has been shown to increase in inclement weather [15,29,32,40,41]. However, some studies have stated contradictory results, claiming that the severity of pedestrian injuries decreases in inclement weather [42,43]. In particular, Yu [44] has argued that both drivers and pedestrians are likely to refrain from going out when the weather is bad, resulting in less serious injuries; Michalaki et al. [43] and Quddus et al. [45] have also claimed that the severity of pedestrian injuries increases under dryer road conditions. Additionally, Aziz et al. [46] also studied factors affecting the severity of pedestrian-vehicle crashes in New York, revealing that pedestrian injuries are more serious under wet road conditions. Lee and Lee [47] demonstrated that weather and road surface conditions differently influence the severity of pedestrian injuries. Based on these studies, it can be concluded that inclement weather may affect the vision of drivers and lengthen the vehicle stopping distances. However, inclement weather may also lead to reduced vehicle speed and generally play a significant role in reducing exposure for pedestrians, as people are less likely to go out walking in bad weather.

Brightness and darkness also have different effects on the severity of pedestrian injuries. Previous studies have emphasized that poor visibility and darkness increase the severity of pedestrian injuries [41,42,48]. However, Quddus et al. [45] also noted that the severity of pedestrian injuries sometimes decreases when it is dark.

Crash event characteristics, such as time of the day [43,49], crashes at intersections or crosswalks [15,47], and a driver’s level of intoxication [37,50], have been considered as determinants influencing the severity of pedestrian injuries. Meanwhile, Zhang et al. [37] exhibited that drunk driving results in more serious pedestrian injuries and argued that punishments for drunk driving should be more stringent.

### 2.2. Neighborhood Environmental Characteristics of Pedestrian-Vehicle Crashes

Various studies have considered neighborhood environmental characteristics that may affect the severity of pedestrian injuries, such as demographic factors, road features, land use, and land development indicators [46,51,52,53,54,55,56].

Studies on demographic factors mainly focus on the age of the drivers and the pedestrians and population density by age [32,36,48,55,56,57]. Some studies have demonstrated that populations with a higher ratio of residents of 65 years and older have more serious pedestrian injuries [32,48,57]. Clifton and Kreamer-Fults [56] showed that pedestrian injuries are more serious in pedestrian-vehicle crashes occurring near schools in densely populated neighborhoods with a large population of children between 5 and 15 years. This finding corresponds with Bae and Park [15]. However, Kim et al. [35] also studied the injury severity of pedestrian crashes across South Korea by employing a hierarchically ordered model, thereby arguing that the higher the population density, the lower the severity of pedestrian injuries. When studying road accidents in Hong Kong, Sze and Wong [48] demonstrated that as the percentage of the population under the age of 15 years increases, pedestrian injuries become less serious. Similar results were reported in Narayanamoorthy et al. [55], that analyzed 285 census tracts from Manhattan, New York. Noland and Quddus [57] also reported similar findings while analyzing the severity of pedestrian injuries in 11 UK regions. Therefore, depending on the study sites and the spatial units, demographic factors have affected pedestrian road fatalities in an inconsistent manner. Even when analyzing the same study site and spatial unit, results may vary depending on the age groups.

Regarding road characteristics, studies have shown that narrow road width and low road density have a negative effect on the severity of pedestrian injuries [32,35,47,56]. These findings have implied that countries with wider roads and higher road density experience more crashes involving pedestrians. However, Kim et al. [35] examined types of roads in more detail and found that the severity of pedestrian injuries was less on roads with a width of less than 3 m. Therefore, in future studies, it is necessary to subdivide the effects of pedestrian risk exposure on the basis of the road width. In general, crosswalks reduce the severity of pedestrian injuries [15,33,39,58]. However, Tay et al. [32] also studied the severity of pedestrian injuries by using a multinomial logit model and found that crosswalks increase the severity of pedestrian injuries. Unlike crosswalks, intersections increased the severity of pedestrian injuries in several studies [19,29,32,48]. In addition, other studies have considered various road characteristics, such as traffic signals [33,37,49], speed limits [59,60,61], and signs [19,50], as factors influencing the severity of pedestrian injuries.

In addition to demographics and road characteristics, land use characteristics have also been considered in studies on pedestrian injury severity [23,55,62,63,64]. Narayanamoorthy et al. [55] analyzed 285 census tracts in New York and noted that residential, commercial, and industrial areas have a similar level of severity of pedestrian injuries, regardless of land use type. Kwon and Park [14] also examined the effects of land use around elementary schools in South Korea and noted that residential areas seem to experience fewer pedestrian fatalities. Kim et al. [31] also studied the severity of pedestrian injuries in North Carolina, U.S., and found that commercial areas are likely to experience less severe pedestrian injuries. However, this finding contradicted with Narayanamoorthy et al. [55]

Development density is also related to the severity of pedestrian injuries [14,36,47]. Mohamed et al. [36] proved that single or double family residential land use decreases the likelihood of pedestrian fatalities in New York. Kwon and Park [14] have also studied safe routes to school in residential areas of Seoul and found that the higher the floor area ratio of the residential buildings around elementary schools, the lower the severity of pedestrian injuries. Lee and Lee [47] have indicated that the gross floor area of commercial facilities reduces the severity of pedestrian injuries. However, they also found that the gross floor area of industrial facilities increased the severity of pedestrian injuries. In addition, various neighborhood environmental factors, such as urban areas [33,38], school zones [15], and parking lots [36,40], have been studied as factors influencing the severity of pedestrian injuries.

This literature review demonstrates that various factors affect the severity of pedestrian injuries. Previous studies have shown that the individual characteristics of the drivers and the pedestrians affect the severity of injuries in pedestrian-vehicle crashes. Additionally, studies have also shown that neighborhood environmental characteristics affect pedestrian-vehicle crashes differently, depending on the study sites and the spatial units. However, in South Korea, little research has been conducted on the severity of pedestrian injuries that consider both the individual characteristics of crashes and the built environment of neighborhoods.

Therefore, this study employs a multilevel model to identify factors influencing the severity of pedestrian injuries, by considering not only the neighborhood environmental characteristics, but also individual crash characteristics.

## 3. Data and Model

### 3.1. Data

**Figure 1:**
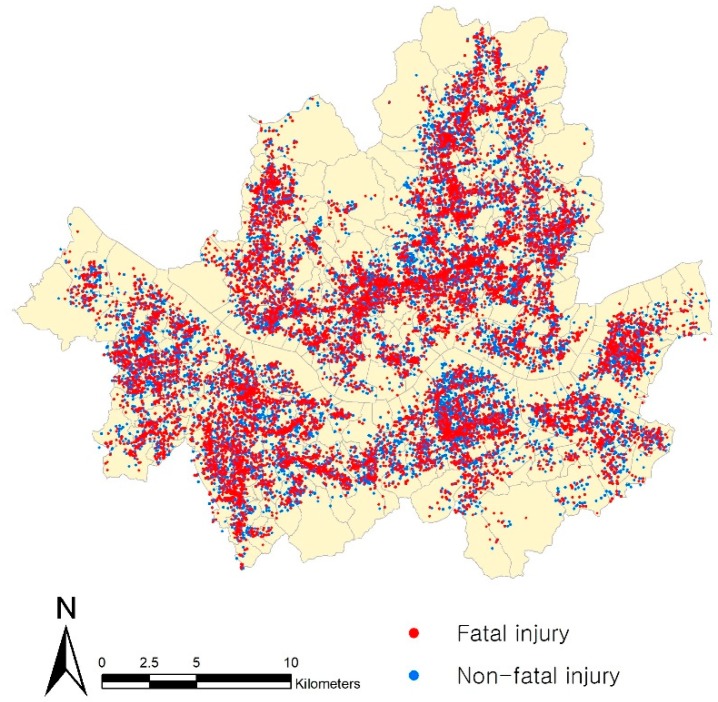
Spatial distribution of pedestrian-vehicle crashes in Seoul.

This study used pedestrian-vehicle crash data from 2015 to 2017 provided by Korea′s Traffic Accident Analysis System [65]. In all, 24,827 pedestrian-vehicle crashes were geocoded through the GIS program (Figure 1). Through a literature review, the lower-level individual characteristics of crashes were divided into three categories; namely, pedestrian, driver, and crash event characteristics. The individual characteristics of crashes include pedestrian age and sex, driver age and sex, vehicle types, weather, crash at intersections, crash at crosswalks, and time variables.

For upper-level neighborhood environmental characteristics, built environment characteristics of 424 administrative districts in Seoul were measured. The neighborhood-level attributes were divided into five characteristics; namely, roads, land use, land development, safety zones, and population demographics.

For road characteristics, road humps, neighborhood streets, main roads, signalized crosswalks, non-signalized crosswalks, signalized intersections, non-signalized intersections, and posted speed were considered. These variables were provided by the Seoul Open Data Plaza [66] and the Road Name Address Guidance System [67]. Land use data provided by the Seoul Urban Planning Portal were used to examine residential and commercial areas [68]. In addition to two-dimensional land use, building data provided by the National Spatial Data Infrastructure Portal were used to measure the average gross floor area of residential buildings and the average gross floor area of commercial buildings to explore the effects of land development on pedestrian-vehicle crashes [69]. In addition, data provided by the Open Data Portal and the Seoul Open Data Plaza were used to evaluate the impact of school zones and silver zones, which represent central transportation policies for pedestrian safety in South Korea [66,70]. Finally, demographic data provided by the Korean Statistical Information Service were used to examine general population density and the population density of those under 15 years and those over 65 years [71].

### 3.2. Estimated Stage of Multilevel Binomial Logistic Model

Individuals are influenced by the characteristics of the built environment in which they live. In addition, individuals belong to larger groups that share common characteristics that distinguish individuals of that group from individuals of another group [72]. A multilevel model is a statistical analysis method for analyzing data with a hierarchical structure composed of individuals and groups [73]. This multilevel model can account for the effects of interactions between levels and the direct effects of variables measured at different levels [74]. This study aims to identify factors that affect the severity of pedestrian injuries, by examining individual characteristics of crashes as lower-level factors and neighborhood environmental characteristics as higher–level factors. The estimation process of a multilevel linear model is given below [35,73,74,75,76].

Step one sets up an intercept-only model. This model is mainly referred to as a null model. The Equation (1) of step one is expressed in the following manner:

(1) $$Y_{ij}=\gamma_{00}+u_{0j}+e_{ij}$$

A null model does not include any explanatory variable and includes only error terms at the upper and lower levels. In a null model, $\gamma_{00}$ is the overall mean of the sample. $u_{0j}$ is the difference of the mean between neighborhoods. $e_{ij}$ is the difference between individual accidents.

Step two sets up a model that applies the individual characteristics affecting a dependent variable as explanatory variables. Equation (2) of step two is expressed in the following manner:

(2) $$Y_{ij}=\gamma_{00}+\gamma_{p0}X_{pij}+u_{0j}+e_{ij}$$

$X_{pij}$ is an explanatory variable applied at a lower level. At the step two, the intercept and the slope coefficient are assumed to vary across neighborhoods (upper level). Step two estimates the influence of explanatory variables that affect the variance of a dependent variable at the lower level. In addition, the change in variance of the lower level and the upper level is estimated by incorporating explanatory variables into the lower level.

Step three expands the model by adding neighborhood environmental characteristics as explanatory variables at the neighborhood level, which is the upper level. The Equation (3) of step three is expressed in the following manner:

(3) $$Y_{ij}=\gamma_{00}+\gamma_{p0}X_{pij}+\gamma_{0q}Z_{qj}+u_{0j}+e_{ij}$$

$Z_{qj}$ is the explanatory variable applied at the upper level. If $Y_{ij}$ is binary, a hierarchical binomial logistic model is appropriate. At step three, the intercept is assumed to vary across neighborhoods, but the slope coefficient is assumed not to vary across the neighborhood (upper level).

The Equation (4) of the multilevel binomial logistic model used to determine the severity of pedestrian injuries in this study is expressed in the following manner:

(4) $$\log\left(\frac{pij}{1-p_{ij}}\right)=\gamma_{00}+\overset{Q}{\underset{q=1}{\sum }}\gamma_{0q}X_{qj}+\overset{P}{\underset{p=1}{\sum }}\gamma_{p0}Z_{pij}+u_{0j}$$

$P_{ij}$ is the pedestrian severity of injury. $\frac{pij}{1-p_{ij}}$ is the odds ratio of a fatal injury. $\gamma_{00}$ is the intercept. $X_{qj}$ is the explanatory variable of individual characteristics of crashes, which include the characteristics of drivers, pedestrians, and crash events. $Z_{pji}$ is the explanatory variable of neighborhood environmental characteristics. $u_{0j}$ is the random effect of neighborhood environmental characteristics, which include the characteristics of roads, land use, land development, safety zones, and population characteristics.

## 4. Results and Discussion

### 4.1. Results of Descriptive Statistic Analysis

Table 1 depicts a descriptive analysis identifying factors that affect the severity of pedestrian injuries in Seoul. In this study, the severity of pedestrian injuries as a dependent variable has an average value of about 0.43; this means that about 43% of pedestrian-vehicle crashes in Seoul result in a fatal injury. In other words, pedestrian safety in Seoul is very poor.

The average ages of a pedestrian and a driver involved in a pedestrian-vehicle crash are 46 and 49 years, respectively. Men and women are involved in pedestrian crashes at similar rates; males account for about 51% of all pedestrians involved in pedestrian-vehicle crashes. However, males account for about 81% of all drivers in pedestrian-vehicle crashes. Despite the increase in the number of female drivers in South Korea each year, men are consistently involved in more pedestrian-vehicle crashes than women. Trucks and vans are involved in 22% of all pedestrian-vehicle crashes. In addition, approximately 12% and 42% of pedestrian-vehicle accidents occurred during inclement weather and at night, respectively, in Seoul. Approximately 32% of pedestrian traffic accidents occurred at intersections, while about 8% of pedestrian-vehicle crashes occurred at crosswalks.

Regarding built environment characteristics, neighborhoods have an average of 22 road humps. Neighborhood streets, which are largely used for pedestrian traffic, account for about 62% of all roads. Main roads, which are mainly used for vehicle traffic, account for about 22%. Neighborhoods have an average of 25 and 52 signalized and non-signalized crosswalks, respectively, and neighborhoods have an average of 48 and 173 signalized and non-signalized intersections, respectively. Non-signalized intersections are three times more likely than signalized intersections to result in pedestrian injury. The average posted speed of neighborhoods in Seoul is approximately 50.48 km/h. On average, about 71% of neighborhoods are residential, while commercial areas are less common, i.e., about 8%. The average gross floor area of residential buildings in a neighborhood, which is an index of residential development, is about 1423 m^2^, and the average gross floor area of commercial buildings in a neighborhood, which is an index of commercial development, is about 1566 m^2^. Each neighborhood has an average of four school zones, which are a part of South Korea’s Safe Routes to School program. Despite South Korea being a rapidly aging society, on average, each neighborhood has only 0.24 silver zones, which are areas meant to provide safe walking environments for the elderly. Finally, the average population density of neighborhoods in Seoul is about 24,705 people per km^2^. The average population density for those under 15 is about 2702 people per km^2^, and the average population density for those above 65 is about 3068 people per km^2^.

### 4.2. Discussion of Findings

Table 2 presents the results after applying a multilevel binomial logistic model. Model 1 is an unconditional model that does not include independent variables. This model only calculates the explanatory power of the probabilities of a fatal injury from the variance data of the individual characteristics (lower level) and neighborhood environmental characteristics (upper level) before the independent variables are included. Model 2 includes only individual characteristics (lower level). Model 3 includes both individual characteristics (lower level) and neighborhood environmental characteristics (upper level). The intraclass correlation coefficient (ICC) values represent the hierarchical explanatory power of both the individual and the neighborhood environmental characteristics [74,75]. The ICC values are 0.023, 0.018, and 0.040 for models one, two, and three, respectively; these findings indicate that the individual characteristics of pedestrian crashes play a significant role in explaining the severity of pedestrian injuries in Seoul. Contrastingly, the impact of characteristics of the built environment on the severity of pedestrian-vehicle crashes in Seoul is relatively low. Therefore, these findings suggest that more attention should be paid to safety awareness and traffic regulations through education and awareness campaigns, to reduce the severity of pedestrian-vehicle crashes. In addition, behavioral problems do not necessarily imply the need for behavioral solutions; for example, in the case of preventing drivers from falling asleep, a reasonable number of rest stops and rumble strips need to be constructed.

However, it cannot be said that the characteristics of the built environment of neighborhoods have no influence on the severity of pedestrian-vehicle crashes. More informed urban planning, urban design, and transportation policies can reduce the likelihood of pedestrian crashes and pedestrian fatalities, by improving neighborhood environments. Hence, it is necessary to not only better educate drivers and pedestrians and strengthen laws, but also to improve the built environments of neighborhoods for pedestrian safety.

An analysis of the lower-level individual characteristics reveals that all variables except a driver’s sex have an influence on the severity of pedestrian injuries. Female pedestrians are more likely to be involved in a pedestrian-vehicle crash than men; this finding is consistent with the results of previous studies [15,32,35].

Similar to previous studies [32,35,36,37,38], it was shown that the older the pedestrian, the more fatal the accident. Older people are less active, and therefore they are physically weak. Demetriades et al. [77] conducted a study on the relationship of age on injury type and severity and demonstrated that the risk of severe pedestrian injury is higher among elderly pedestrians. South Korea has a rapidly aging population, and therefore reducing injuries to elderly pedestrians urgently needs to be addressed.

Additionally, the severity of pedestrian injuries tends to be more serious as drivers get older; this finding also agrees with earlier studies [15,78].

Jang et al. [79] stated that elderly drivers have a higher severity of pedestrian injuries due to a lack of judgment, slower reaction speed, and poor vehicle control when accidents occur. Therefore, as the population ages, it is necessary to increase the visibility of road signs and install better streetlights to improve nighttime visibility. There also prevails a need for specialized traffic safety programs targeted toward the elderly, such as the silver mark system. A driver’s license renewal system for the elderly is also advisable [80]. In addition, there prevails a need for various incentive systems, such as providing vouchers to elderly drivers in return for relinquishing their driver’s licenses voluntarily.

Similar to previous studies, the severity of pedestrian-vehicle crashes were higher among trucks and vans [23,31,36,37,38,39]. Therefore, special training and education must be included in truck and van driving tests, to strengthen pedestrian safety.

As shown in Table 1, the incidence of pedestrian-vehicle crashes in inclement weather is only 12%. However, pedestrian injuries suffered in inclement weather are more serious; these findings agree with previous studies [15,20,32,39,40,41]. In contrast to these findings, Yu [44] has studied pedestrian-vehicle crashes in 140 census tracts in Austin, Texas and found that pedestrians and drivers tend to stay at home during inclement weather, reducing the severity of pedestrian injuries. Therefore, in the case of inclement weather, smart transportation facilities incorporating networked devices need to be developed, to make it easier for drivers to recognize pedestrians from afar.

The results of the model also show that pedestrian-vehicle crashes are more likely to occur at night. Mohamed et al. [36] demonstrated a similar result when studying New York and Montreal. He asserts that, at night, both drivers and pedestrians do not have enough time to react in the event of a sudden crash, due to low visibility. Hence, it is necessary to improve overall nighttime visibility on roads, particularly in places where crashes frequently occur.

Pedestrian-vehicle crashes occurring at crosswalks or intersections are more likely to result in serious injury; this finding agrees with previous studies [15,29,47,48]. Intersections and crosswalks are generally spots where drivers concentrate on driving safety. Nevertheless, the high severity of pedestrian injuries occurring at intersections and crosswalks highlights the need for greater attention to pedestrian safety around intersections and crosswalks. At intersections, drivers are often distracted. Similarly, pedestrian injuries often result from a driver unable to see a pedestrian. For accidents at crosswalks, drivers may continue to drive without noticing that the traffic signal has changed. Contrastingly, pedestrians may fail to see oncoming traffic or ignore a signal while jaywalking. Therefore, both drivers and pedestrians need to be educated about how to drive and walk safely around intersections and crosswalks. However, a few studies state that crashes at intersections have a lower injury severity compared to other pedestrian-vehicle crashes [36,81]. These studies assert that traffic at intersections is more complicated, and therefore drivers may voluntarily slow down their vehicles.

Interestingly, among various neighborhood environmental characteristics, land use, land development, the prevalence of safety zones, and population demographics are not statistically significant for explaining the severity of pedestrian injuries. In fact, only road characteristics play a significant role in explaining the association between the built environment and injury severity. As noted above, it seems that lower-level individual characteristics have a greater influence on the severity of pedestrian injuries than the built environment. However, it is important to consider the contextual effects of each characteristic, even if there is no statistical significance for some built environment characteristics.

Among road characteristics, road humps have been shown to increase the severity of pedestrian-vehicle crashes. However, previous studies have shown that road humps can effectively reduce the vehicle speed, and therefore, the frequency and severity of pedestrian-vehicle crashes [82,83]. According to a survey on the safety of road humps by the Korea Consumer Agency, more than 60% of humps did not meet installation standards in 2014 [84]. Therefore, improperly installed and maintained road humps that do not meet the installation standards may threaten the safety of the pedestrians and the drivers in South Korea.

There is no statistically significant correlation between the severity of pedestrian injuries and road hierarchy. Neighborhood streets mainly used by pedestrians appear to be poorly associated with the severity of pedestrian injuries at a level of *p* < 0.1; this finding is in agreement with previous studies that indicate that neighborhood streets experience a lower number of crashes that result in severe pedestrian injuries [32,35,47]. However, main roads, which are primarily used for vehicle traffic, are not statically associated with a higher incidence of severe pedestrian injuries. Even more interestingly, posted speed also does not show a statistical significance. However, several studies have revealed that posted speed is likely to increase the severity of pedestrian injury [15,38,39,43,47]; this counterintuitive finding implies that, in these situations, crashes occur because of increased speed rather than road hierarchy or posted speed. In addition, the existence and condition of sidewalks where pedestrians can walk safely also needs to be considered as an important factor.

This study has revealed new insights into pedestrian-vehicle accidents at crosswalks and intersections. The majority of previous studies have not studied signalized crosswalks and non-signalized crosswalks separately [15,33,39,58]. Instead, these studies have simply indicated that crosswalks play a role in reducing pedestrian injuries. This study divided crosswalks into signalized crosswalks and non-signalized crosswalks and found that, in relation to predicting the severity of pedestrian injury, the presence of a signalized crosswalks did not produce a statistically significant difference, whereas non-signalized crosswalks were observed to increase the incidence of pedestrian-vehicle crashes. In other words, crosswalks without traffic signals were found to be safer for pedestrians than crosswalks with traffic signals.

A similar result was found for intersections. Several studies have shown that intersections generally experience a high number of severe pedestrian-vehicle crashes [19,29,32,48]. However, these studies do not consider differences between intersections with and without traffic signals. In this study, the intersection variable was divided into signalized and non-signalized intersections as well as crosswalks. Interestingly, the study revealed that pedestrian-vehicle crashes that occur at intersections without traffic signals result in less severe pedestrian injuries than pedestrian crashes at intersections with traffic signals. Various factors, such as the vehicle speed, traffic volume, and the number of lanes, can cause significant differences between signalized intersections and non-signalized ones. A possible explanation for the behavior of drivers and pedestrians is that both tend to be more cautious of avoiding crashes at intersections and crosswalks without traffic signals than at intersections and crosswalks with traffic signals. A possible conclusion that can be drawn from these findings is that traffic signals may not contribute significantly to pedestrian safety. Previous studies that examined the relationship between traffic signals and pedestrian–vehicle crashes have yielded conflicting results. Kim et al. [23] and Kim et al. [31] noted that traffic signals improve pedestrian safety, while Celik and Oktay [49] emphasized that traffic signals have a negative impact on pedestrian safety. This study also finds that traffic signals do not necessarily guarantee improved pedestrian safety. This aspect indicates the need for both the drivers and the pedestrians to comply with traffic signals, particularly in South Korea. Hence, there prevails a need to educate the drivers and the pedestrians to ensure that they comply with traffic rules.

The land use characteristics of certain areas in Seoul, whether residential or commercial, were not significantly associated with the severity of pedestrian injuries. A possible explanation for this finding is that residential and commercial areas are not as clearly demarcated as in North America due to the distinctive characteristics of land use in South Korea. Indeed, land use in Seoul is mixed, with commercial facilities such as convenience stores and restaurants commonly appearing within residential areas.

In addition to a two-dimensional concept of land use, this study also examined the characteristics of land development, which refers to the degree of actual development in a three-dimensional setting. Both the average gross floor area of residential buildings and the average gross floor area of commercial buildings had no correlation with the severity of pedestrian injuries. However, higher land development, whether commercial or residential, could correlate to more walking by residents; this aspect suggests that more pedestrians are exposed to possible collisions with vehicles on roads. Therefore, the implementation of transportation policies and strategies such as traffic calming to control traffic volume and traffic flow should be considered in areas with high residential development.

School zones and silver zones, which represent two important transportation policies for pedestrian safety in South Korea, have no statistically significant relation to pedestrian injuries. In South Korea, school zones and silver zones are designated as protection areas on roads within a radius of 300 m. These zones are meant to protect children and the elderly [85]. In particular, school zones are related to the Safe Routes to School policy, which has mainly been studied in North America. However, both of these zones display a negative sign, despite no significance. There are a few studies on the effects of built environment characteristics around school zones on the frequency of pedestrian crashes [14,86]. However, there is little research on the effectiveness of school zones for the severity of pedestrian injuries. School zones in South Korea mainly involve installing and managing traffic safety facilities, such as safety fences, safety signs, and speed cameras. Therefore, there is a need for constant attention concerning the effectiveness of school zones for children to walk safely to school. In addition, it is suggested that children in South Korea need to be taught safe walking skills in kindergartens, and they should practice those skills walking to school in groups. Even the junior high students need to learn how to use the transit system and navigate other neighborhoods. Silver zones also have no statistically significant relationship with pedestrian injuries; however, they demonstrate a positive direction concerning the severity of pedestrian-vehicle crashes; this aspect suggests that silver zones have not yet been institutionalized in South Korea. School zones were first introduced in South Korea in 1995, while silver zones were first introduced in 2006. As of 2017, Seoul has 1733 and 118 school zones and silver zones, respectively [87]. In the future, in addition to school zones, there is a need for continuous attention and research on the efficacy of silver zones for the elderly, who are more vulnerable to traffic accidents.

Finally, the demographic factors according to age groups do not exhibit statistical significance. Although population density is not associated with the severity of pedestrian injury, it indicates a negative direction; this finding is in agreement with previous studies analyzing the same study area in South Korea [35]. However, a study on pedestrian crashes near public schools in Baltimore, US insists that the higher the population density, the higher the severity of pedestrian-vehicle crashes [56].

The population density of those 15 years and younger and the population density of those 65 years and older also have no statistical significance. However, they exhibit a positive direction, which is in contrast to the negative direction of the overall population density. In South Korea, people aged 15 years and younger and people aged 65 years and older are classified as vulnerable road users according to the Act on Promotion of Transportation [88]. This finding suggests that more attention should be given to vulnerable road users who are at risk of being injured in pedestrian-vehicle crashes.

## 5. Conclusions

Countries must provide safe walking environments to provide a high quality of life to their citizens. Therefore, it is important to reduce the frequency of pedestrian-vehicle crashes and the severity of pedestrian injuries. This study investigated built environment characteristics affecting the severity of pedestrian injuries in South Korea, a country with a high rate of pedestrian-vehicle crashes. In particular, this study examined the individual characteristics of pedestrian crashes that have not been considered in previous studies [14,47,89,90]. Previous studies have only examined built environment characteristics, without incorporating individual characteristics of pedestrian-vehicle crashes, and therefore, it has been difficult to identify the effect of built environment characteristics on injury severity accurately. Thus, it can be difficult to develop accurate and effective policies for ensuring pedestrian safety. This study, therefore, employed a multilevel analysis to examine the influence of lower-level individual characteristics of pedestrian crashes, alongside the upper-level built environment characteristics of neighborhoods on the severity of pedestrian injuries. The main results of this study are as follows.

First, the individual characteristics of pedestrian-vehicle crashes are better at explaining pedestrian injury severity than built environment characteristics at the neighborhood level. Therefore, the individual characteristics of pedestrian crashes must be considered in future research on pedestrian-vehicle crashes. In addition to improving the physical environment to increase pedestrian safety, policies that consider attributes of drivers, pedestrians, and crash events are needed.

Second, older pedestrians and drivers suffer more severe pedestrian injuries. Therefore, whether drivers or pedestrians, transportation safety policies and strategies to safeguard the elderly must be developed. It is necessary to expand traffic safety facilities that can ensure the safety of elderly pedestrians. For older drivers, policies that reinforce driving tests and license renewal systems, while also encouraging elderly drivers to voluntary relinquish their driver′s licenses are necessary.

Third, larger vehicles such as trucks and vans are more likely to result in a high severity of pedestrian injuries. Therefore, special safe driving training should be facilitated to large vehicle drivers.

Fourth, pedestrian injuries increase during inclement weather and at night. Therefore, various pedestrian safety facilities and streetlights to increase visibility should be installed. In addition, during bad weather or at night, regulations to lower the posted speed are necessary.

Fifth, pedestrian-vehicle crashes at crosswalks and intersections result in more severe pedestrian injuries. However, the severity of pedestrian injuries is lower at intersections and crosswalks without traffic signals than at crosswalks and intersections with traffic signals. Therefore, qualitative environmental improvements around crosswalks and intersections and quantitative increases of crosswalks and intersections should be implemented, to reduce the severity of pedestrian injury. In addition, it is necessary to consider ways to install roundabouts that can reduce traffic accidents.

Finally, school zones and silver zones, which are representative policies for pedestrian safety in South Korea, fail to play a significant role in reducing the severity of pedestrian injuries. Therefore, in-depth policy development and discussions about the designation of safety zones and the installation of effective traffic safety facilities around safety zones should be implemented and held, respectively, by transportation, urban planning, and urban administration experts.

This study also had some limitations. For example, pedestrian-vehicle crashes are events that occur in open spaces and therefore possess spatial attributes. However, this study was unable to consider spatial dependence and spatial autocorrelation between pedestrian crashes. Future studies should investigate spatial heterogeneity between neighborhoods. Traffic volume and pedestrian volume may also be the contributing factors that directly or indirectly affect the severity of pedestrian injuries. However, this study fails to consider these two potential indicators because of the limitation of the acquired data. More in-depth investigations could be conducted if the necessary data were acquired. Nevertheless, the results of this study can guide policymakers and planners when making decisions on how to build neighborhoods that are safer for pedestrians.

## Figures and Tables

**Table 1:** Descriptive statistics.

Level	Variables		Definition	Unit	Mean	Std. Dev.
Severity of pedestrian injury			Severity of pedestrian injury (1 = Fatal injury, 0 = Other)	Binomial logit	0.43	0.49
Individual characteristics (Lower level)	Pedestrian characteristics	Pedestrian age	Pedestrian age	Number	45.72	20.79
		Pedestrian sex	Pedestrian sex (1 = Male, 0 = Female)	Dummy	0.51	0.50
	Driver characteristics	Driver age	Driver age	Number	49.26	13.38
		Driver sex	Driver sex (1 = Male, 0 = Female)	Dummy	0.81	0.39
	Crash event characteristics	Vehicles	Vehicle types (1 = Truck or Van, 0 = Passenger car)	Dummy	0.22	0.41
		Weather	Weather condition (1 = Inclement, 0 = Other)	Dummy	0.12	0.33
		Time	Time (1 = Night (18–06), 0 = Other)	Dummy	0.42	0.49
		Crash at intersection	Pedestrian-vehicle crash at intersection (1 = At intersection, 0 = Other)	Dummy	0.32	0.46
		Crash at crosswalk	Pedestrian-vehicle crash at crosswalk (1 = At crosswalk, 0 = Other)	Dummy	0.08	0.27
Neighborhood environmental characteristics (Upper level)	Road characteristics	Road humps	Number of humps per km^2^	Density	21.52	18.31
		Neighborhood streets	Proportion of neighborhood streets	Ratio	61.98	16.30
		Main roads	Proportion of main roads	Ratio	21.56	13.12
		Signalized crosswalks	Number of signalized crosswalks per km^2^	Density	25.37	13.49
		Non-signalized crosswalks	Number of non- signalized crosswalks per km^2^	Density	52.11	29.77
		Signalized intersections	Number of signalized intersections per km^2^	Density	47.50	28.04
		Non-signalized intersection	Number of non- signalized intersections per km^2^	Density	172.60	108.84
		Posted speed	Average posted speed	Avg (km/h)	50.48	4.41
	Land use characteristics	Residential areas	Proportion of residential areas	Ratio	70.62	28.47
		Commercial areas	Proportion of commercial areas	Ratio	7.92	15.08
	Land development characteristics	Gross floor area of housing buildings	Average gross floor area of housing buildings	Avg (m^2^)	1423.06	2685.19
		Gross floor area of commercial buildings	Average gross floor area of commercial buildings	Avg (m^2^)	1566.66	2758.04
	Safety zone characteristics	School zones	Number of school zones per km^2^	Density	3.67	2.63
		Silver zones	Number of silver zones per km^2^	Density	0.24	0.51
	Population characteristics	Population	Population per km^2^	Density	24,705.37	16,803.56
		Under 15 years	Population under 15 years old per km^2^	Density	2701.67	2168.02
		Over 65 years	Population over 65 years old per km^2^	Density	3067.75	2170.37

**Table 2:** Results of multilevel binomial logistic model.

Classification		Variables	Model 1			Model 2			Model 3		
			Coefficient	Std. Error	Odds Ratio	Coefficient	Std. Error	Odds Ratio	Coefficient	Std. Error	Odds Ratio
Intercept			−0.201 ***	0.019	0.817	−1.613 ***	0.069	0.199	−1.608 ***	0.291	0.200
Individual characteristics (Lower level)	Pedestrian characteristics	Pedestrian age				0.023 ***	0.000	1.023	0.024 ***	0.000	1.024
		Pedestrian sex				−0.288 ***	0.027	0.749	−0.268 ***	0.029	0.764
	Driver characteristics	Driver age				0.003 ***	0.001	1.003	0.003 ***	0.001	1.003
		Driver sex				0.001	0.036	1.001	0.007	0.039	1.007
	Crash event characteristics	Vehicles				0.133 ***	0.033	1.142	0.132 ***	0.036	1.141
		Weather				0.119 ***	0.040	1.126	0.125 ***	0.043	1.043
		Time				0.278 ***	0.028	1.320	0.286 ***	0.033	1.331
		Crash at intersection				0.216 ***	0.029	1.241	0.247 ***	0.031	1.280
		Crash at crosswalk				0.517 ***	0.050	1.676	0.521 ***	0.055	1.683
Neighborhood environmental characteristics (Upper level)	Road characteristics	Road humps							0.003 ***	0.001	1.003
		Neighborhood streets							−0.003 *	0.002	0.997
		Main roads							−0.002	0.002	0.998
		Signalized crosswalks							−0.002	0.000	0.998
		Non-signalized crosswalks							−0.002 ***	0.000	0.998
		Signalized intersections							0.002 *	0.001	1.002
		Non-signalized intersections							−0.000 ***	0.000	1.000
		Posted speed							0.006	0.004	1.006
	Land use characteristics	Residential areas							−0.000	0.000	1.000
		Commercial areas							0.000	0.001	1.000
	Land development characteristics	Gross floor area of housing buildings							0.000	0.000	1.000
		Gross floor area of commercial buildings							0.000	0.000	1.000
	Safety zone characteristics	School zones							−0.000	0.008	1.000
		Silver zones							0.013	0.034	1.013
	Population characteristics	Population							−0.000	0.000	1.000
		Under 15 years							0.000	0.000	1.000
		Over 65 years							0.000	0.000	1.000
Random Intercept			0.0776			0.0590			0.2683		
ICC			0.023			0.018			0.040		
Deviance			33816.3			32088.4			32033.8		
Number of observations			24826			24826			24826		

*** *p* < 0.01, * *p* < 0.1.
